# Ground-Level Ozone as Community-Acquired Pneumonia Risk Factor in Different Population Groups in Summer: The Case of Moscow

**DOI:** 10.3390/toxics14010083

**Published:** 2026-01-16

**Authors:** Nina Dudorova, Boris Belan, Sergey Kotel’nikov

**Affiliations:** 1 V.E. Zuev Institute of Atmospheric Optics of Siberian Branch of the Russian Academy of Sciences, 1, Academician Zuev Square, Tomsk 634055, Russia; 2 Prokhorov General Physics Institute of the Russian Academy of Sciences, 38, Vavilova Street, Moscow 119991, Russia

**Keywords:** O_3_, ground-level ozone, air pollution, health, age, gender, morbidity, community-acquired pneumonia

## Abstract

A correlation between the near-surface ozone concentration in the urban atmosphere and hospitalizations of community-acquired pneumonia patients has been analyzed based on a long-term (five years) series of observations in the warm season in Moscow, Russia. The study included hospitalization records for patients over 15 years old. One of the main goals was to reveal vulnerable groups of the urban population that react most strongly to increased ozone concentrations. It has been shown that increased near-surface ozone concentrations lead to increased hospitalizations. Older people (over 60 years old) are most sensitive to the negative impact of air pollution. Women in this age group are more sensitive to the effects of ozone air pollution than men. In the middle-aged group (31–60 years), the highest correlation between the number of community-acquired pneumonia cases and the ozone level in the atmospheric surface layer, conversely, was in men, but it was still lower than the rate in older people. The young people (15–30 years old) group turned out to be insensitive to the near-surface air pollution.

## 1. Introduction

Air pollution is one of key global risk factors for premature mortality [1]. Tropospheric ozone is among main components of urban smog; it is a secondary photochemical pollutant, and a strong oxidant. As a gaseous pollutant, ozone primarily affects the human lungs. Long-term exposure to ozone resulted in approximately half a million deaths worldwide in 2021 [2]. According to World Health Organization (WHO) recommendations [3], the air quality standard for ground-level ozone is the daily maximum 8 h average ozone concentration (O_3__MDA8) equal to 100 μg/m^3^.

Adherence to the air quality guideline (AQG) levels of harmful substances in the atmosphere supports health. However, a number of studies have shown that inhalation of just a little higher ozone concentrations can result in such respiratory symptoms as lung function decrements [4,5], epithelial damage [6,7], airway hyperreactivity [8] and inflammatory reactions [9]. Moreover, exacerbation of asthma [10,11,12] and chronic obstructive pulmonary disease (COPD) [13,14] is usually observed.

Pneumonias are a group of acute infectious diseases, primarily of bacterial origin, that vary in their etiology, pathogenesis, and morphological features. These conditions are characterized by focal lesions of the respiratory regions of the lungs, accompanied by the obligatory accumulation of intra-alveolar exudate [15]. Community-acquired pneumonia (CAP) is defined as a disease that develops outside a healthcare facility or within the first 48 h of hospitalization. CAP is one of the most common acute infectious diseases. According to official statistics, the CAP morbidity rate worldwide ranges from 150 to 1400 cases per 100,000 adults per year and depends on geography, season, and population characteristics. In the United States, the morbidity rate is 248 cases per 100,000 adults per year [16]. The total annual average CAP morbidity rate is 187 cases per 100,000 people in China [17]. It was 433.8 cases per 100,000 people in the Russian Federation in 2005–2017 [18]. In terms of age, elderly people over 65 years old are 10 times more likely to develop CAP than adults younger 65 [19]. In the structure of morbidity, the share of pneumonia of all respiratory diseases is small. However, it accounted for up to 50% in the structure of mortality from respiratory diseases in the Russian Federation for the same period [20].

CAP is caused by various microorganisms, including viruses, bacteria, fungi, and protozoa. Numerous risk factors associated with the development of CAP are discussed in the literature. These include conditions such as human immunodeficiency virus infection, behaviors like smoking and excessive alcohol consumption, a low body weight, frequent interaction with children, inadequate oral hygiene, substance abuse, and a sedentary lifestyle, among others [19,21,22,23]. Nevertheless, there is a limited number of studies investigating the effects of air pollution, specifically ozone, on the morbidity rate of CAP, and the findings remain inconclusive. Li et al. [24] demonstrated the effects of air pollutants on acute respiratory conditions among outpatients. Their findings indicated that exposure to O_3_ was associated with an increase in outpatient visits for asthma exacerbations, while it showed a decrease in visits for acute respiratory viral infections, CAP, and bronchiectasis exacerbations. In this regard, it can be argued that the study of the impact of ground-level ozone on human health is a relevant task.

The aim of this work is to assess the impact of ground-level ozone on CAP morbidity rate in different gender and age groups of residents of a megapolis over a long time. The main feature of this study is the analysis during the summer period, in the absence of seasonal outbreaks of diseases, as well as the selection of a region with comfortable air temperatures, which reduces the impact of a number of other factors on human health.

## 2. Materials and Methods

The study was performed for Moscow and the Moscow suburbs over a five-year period (2006–2009 and 2011). We excluded 2010 from the study because of unprecedented air pollution in Moscow with smoke from wildfires caused by extremely high air temperatures. The relationship between air pollution with ozone and the number of CAP cases in the summer of 2010 is studied in detail in [25]. Ozone concentrations were measured every 20 min at four Mosecomonitoring stations [26]: Maryino, Zelenograd 16, Spiridonovka, and Zvenigorod. Despite the stations being located in the central areas of the city and its outskirts with different landscapes and local population densities, the measurements were in good agreement (the correlation coefficients for ground-level ozone concentrations in summer ranged from 0.8 to 0.95 for all stations used). Based on the data from each station, 8 h average ground-level ozone concentrations were calculated by the moving average method, and the daily maxima (O_3__MDA8) were selected from them. The calculated values were averaged over the four stations, thus providing Moscow-wide average maxima of 8 h average ozone concentrations.

**Figure 1:**
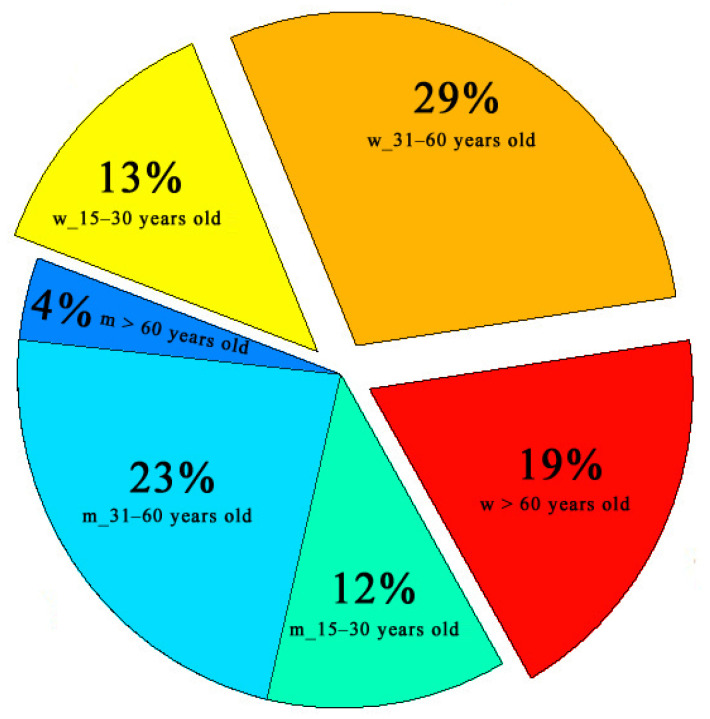
Distribution of Moscow population by gender and age as on 1 January 2011 [18].

To analyze the number of CAP cases, we used data provided by A.S. Puchkov Emergency and Urgent Medical Care Station of the Moscow Department of Health [27]. The Station combines a network of 60 substations and 42 posts uniformly distributed over the city. According to statistical information obtained from the data source, the difference between preliminary and final diagnoses for the class of diseases examined is less than 10%. Therefore, we suppose that preliminary diagnoses can be used for analysis. The database of diagnosed CAP cases was divided into gender groups (men and women), and each group was divided into three age subgroups: (1) 15–30 years old, (2) 31–60 years old, and (3) 60 and over. The number of Moscow residents in each age subgroup is given in Figure 1. The figure shows that there are 22% more women than men. One can see the close share of men and women in the first age subgroup, 6% more women than men in the second subgroup, and 15% more women than men in the third subgroup. In addition, the total number of Moscow residents aged 15 and over increased by 7.8% for the five years under study [18]. This information was taken into account for correct estimates of the interannual variability of the number of CAP cases and the morbidity in groups of men and women of the same age.

As is known, there is a time lag between air pollution peaks and the response in human health, which ranges from one day to several days. To minimize the statistical error, the series of ground-level ozone concentrations and CAP cases were averaged over few days by the moving average method.

## 3. Results

### 3.1. Characterization of the Period and Population Groups Under Study

**Figure 2:**
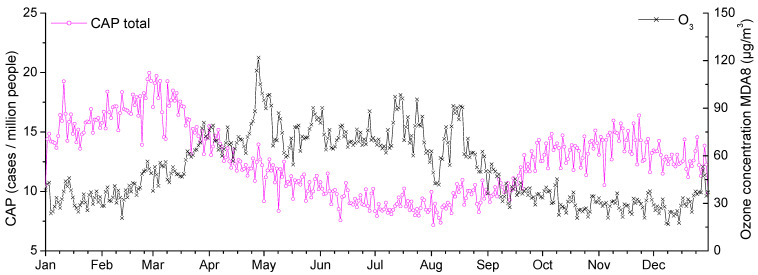
Annual variations in the ground-level ozone concentration (black) and the number of CAP cases normalized to a million Moscow residents aged 15 and over (violet), and averaged over the period under study.

Figure 1 shows the annual variations in the ground-level ozone concentration and the total number of CAP cases normalized to a million Moscow residents aged 15 and over averaged over the period under study. It is evidently impossible to analyze the correlation between ground-level ozone and CAP over a long time, such as a year or even individual seasons, because of their almost opposite annual variations, which result in a false-positive or false-negative correlation. The only season when this study makes sense is summer, when the number of CAP cases is minimal, the seasonal trend shows no obvious increase or decrease, and the ground-level ozone concentrations are maximal. In other seasons, the effect of air pollution on the CAP morbidity rate is not so significant, although a short-term increase in the number of CAP cases with the ozone concentrations is seen even in the averaged data series (Figure 2). In this work, the effect of ground-level ozone on the CAP morbidity rate in summer months is studied.

Table 1 presents ground-level ozone concentrations (O_3__MDA8), maximum daily temperature (t_max_), and the number of CAP cases averaged over June–August of each year.

The average values of O_3__MDA8 were lower than the maximum permissible concentration (MPC) of ozone. The air temperature and air pollution were maximal in 2011. The air temperature and ground-level ozone concentrations were much lower in the summers of 2006, 2008, and 2009 than the averages over the period under study. The parameters were close to the averages over the period under study in the summer of 2007.

**Figure 3:**
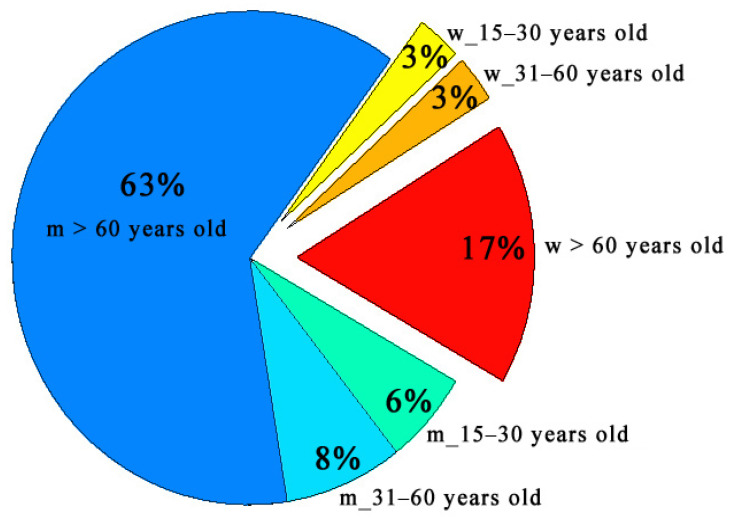
Percentage of CAP cases of the total number of cases over the summer seasons (2006–2009 and 2011) on average in different subgroups considering the number of people in each group.

First, it was interesting to study the percentage of CAP cases of the total number of cases over the summer seasons (2006–2009 and 2011) on averages in different subgroups considering the number of people in each group and neglecting the impact of air pollution (Figure 3). It was shown that men are three times more likely to suffer from CAP than women. Moreover, the main share of CAP cases occurs in the third group (60 years and over). In other age subgroups, the percentage was 3–8% of the total number of pneumonias. These data are in good agreement with the results published in the literature. For example, Torres et al. [19] studied risk factors of CAP in adults in Europe and showed a tenfold increase in CAP morbidity rate in the elderly 65 years and over relative to younger population and higher CAP morbidity rate in men.

### 3.2. Effect of Ground-Level Ozone on the Development of CAP in Different Subgroups

**Figure 4:**
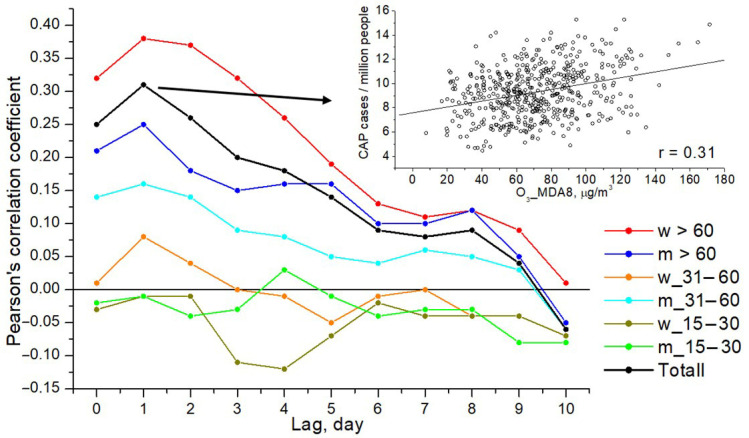
Time dependence of the cross-correlation coefficient between CAP and ground-level ozone concentration. Top right: total number of CAP cases versus ground-level ozone concentration (CAP values correspond to the day following the ozone concentration measurement, i.e., a 1-day lag).

Figure 4 shows the time dependence of the cross-correlation coefficients of CAP and ground-level ozone concentration for the various population groups noted above. It is evident that, on average, the response of human health to an increase in ozone concentration is 1–2 days. All correlation coefficients greater than 0.15 reached a significance level of 0.001 due to the large sample size of 515 cases. For one of the points corresponding to a 1-day lag in the health response to an increase in ozone concentration, the distribution of the total number of CAP cases versus ground-level ozone concentration is presented. A relationship between the studied variables is evident. It can be noted that the relatively low values of the correlation coefficients are associated with statistical error caused by significant daily fluctuations in ozone concentration (fluctuations are evident even in the values averaged over 5 years, presented in Figure 2) and the different durations of the health response of different people. To eliminate this statistical error, the averaging (using the moving average method) of the time series of CAP cases and ground-level ozone concentration over three days was performed. Next, the results are presented for the averaged data.

**Figure 5:**
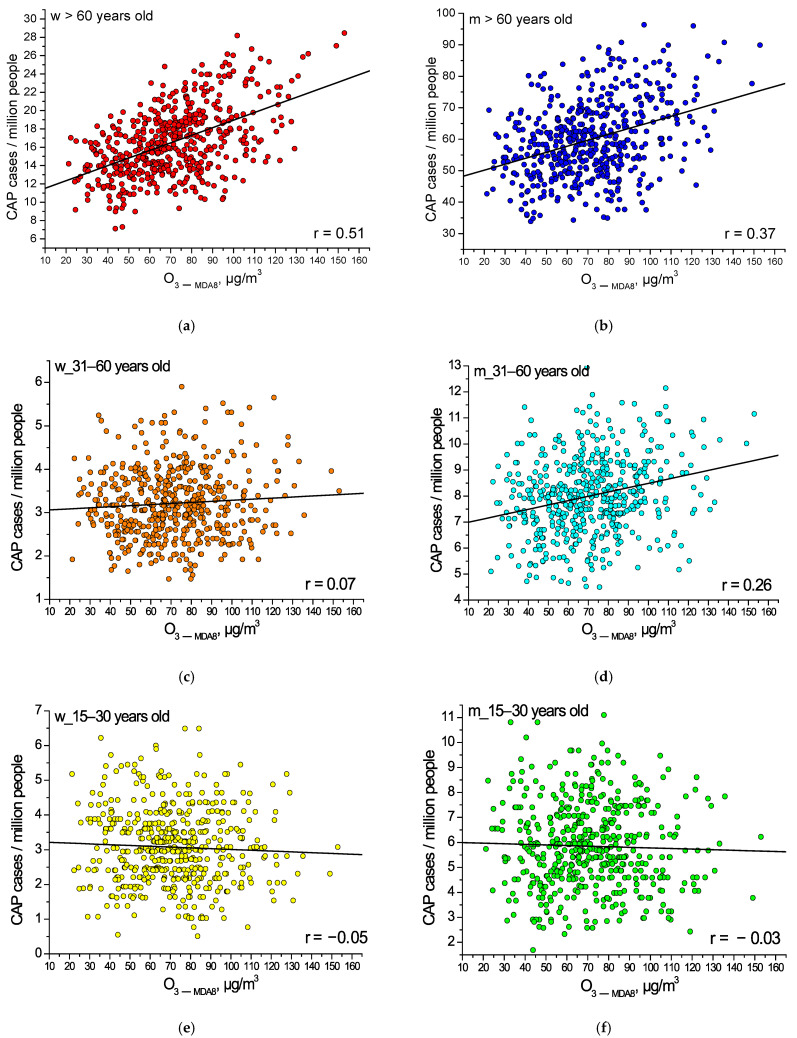
The number of CAP cases versus the ground-level ozone concentration in different population groups (averaged over 3 days, with a 1-day lag): (**a**) women aged 60 years and over; (**b**) men aged 60 years and over; (**c**) women aged 31–60 years old; (**d**) men aged 31–60 years old; (**e**) women aged 15–30 years old; (**f**) men aged 15–30 years old.

Let us analyze which population groups are most vulnerable to an increase in the ground-level ozone concentration. Figure 5 shows the dependence of the number of CAP cases (per million people) on the ground-level ozone concentration in different subgroups over the period under study. One can see from Figure 4 and Figure 5 that the elderly are most susceptible to the impact of air pollution; elderly women are more susceptible to ozone air pollution than elderly men. As the ground-level ozone concentration increases above 100 μg/m^3^, the number of CAP cases among women of aged 60 and over increases by an average of 22% as compared to lower ozone concentrations, and by an average of 14% among men in this age group. In the age group 31–60 years old (Figure 5c,d), an increase in the number of CAP cases with the ground-level ozone concentration was found only for men, but less pronounced than for older people. The number of CAP cases increases by an average of 10% with an increase in the ozone concentrations above 100 μg/m^3^ in the group of men aged 31–60 years old. Young people, 15–30 years old, do not react to an increase in the ground-level ozone concentrations (Figure 5e,f).

**Figure 6:**
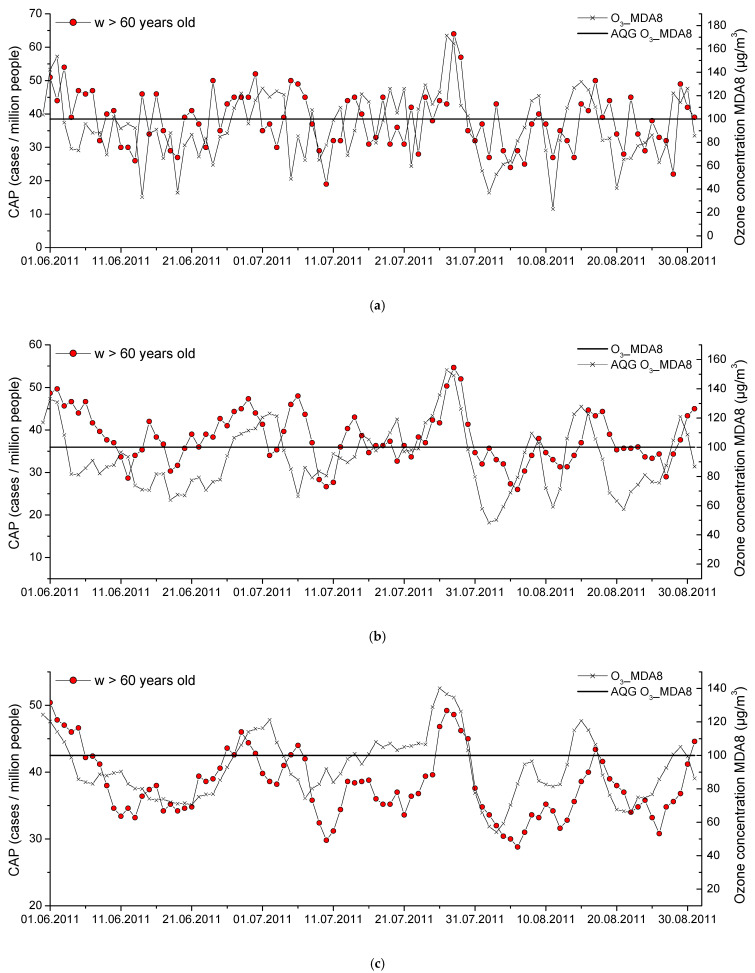
Ground-level ozone concentrations in comparison with the number of CAP cases among women aged 60 years and over in the summer of 2011. Time series without averaging (**a**), averaging over 3 days (**b**), averaging over 5 days (**c**).

Figure 6 shows the seasonal variations in the ground-level ozone concentration and the number of CAP cases for the group of women aged 60 years and over in the summer of 2011, the season with the highest air pollution over the period under study. There is a slight time delay of 1–2 days between the peaks of the CAP and the ground-level ozone concentration. When the averaging period of the CAP and ground-level ozone concentration time series is increased to 5 days one can see similar behavior for both curves for elderly women. Because in this case we are not only talking about high ozone concentrations (more than 100 μg/m^3^) we can conclude that the deterioration of the health of elderly women is possible at lower ground-level ozone concentrations than those accepted by the WHO.

High ozone concentrations are known to correlate strongly with high ambient temperatures and solar radiation. Heat stress is a known risk factor for respiratory distress and hospitalization in the elderly. Table 2 presents the correlation coefficients of 3-day averaged temporal CAP with O_3__MDA8 and air temperature. Clearly, the correlation between CAP and temperature is significantly weaker than the correlation with ozone. This suggests that the increase in community-acquired pneumonia cases is probably caused by ozone, rather than simply heat.

## 4. Discussion

In this study, we examined the relationship between ground-level ozone pollution and hospitalizations for community-acquired pneumonia in Moscow, Russia. Our results show that elevated ground-level ozone levels are associated with increased hospitalizations for pneumonia. On average, regardless of patient age and gender, at ground-level ozone levels of 100–125 μg/m^3^, the incidence of community-acquired pneumonia increases by more than 10% compared to periods with low concentrations. The adverse effects of ozone on the human respiratory system are plausible in real-life conditions. It has been established that ozone can damage tissues and cause oxidative modification of proteins, which, in turn, can decrease the protective function of lungs and increase susceptibility to respiratory infections [28]. The experiment with mice conducted by Mikerov et al. [29] showed an increase in the oxidation of surfactant protein A in epithelial lining fluid in lungs after ozone inhalation, which decreased the immune protection and increased the sensitivity of the mice to experimental pneumonia. Evstaf’ieva et al. [30] found stable positive correlations between high AQG levels and bronchial asthma exacerbation in all seasons in Simferopol. Stepanov et al. [31] showed a noticeable correlation between the ground-level ozone concentration and COVID-19 cases and mortality. Krivosheev et al. [32] proved that ozone had a positive effect on the dynamics of the COVID-19 pandemic as a disinfectant against airborne viruses, and an increase in its concentration was accompanied by a decrease in the saturation of the surface air layer with the viruses. However, ozone was a negative factor for SARS-CoV-2-infected persons, and an increase in the ground-level ozone concentrations ultimately increased the morbidity and mortality rates. Also, no significant association between ozone concentrations and respiratory diseases was observed in the work of A. C. Miranda et al. [33].

An analysis of Moscow population subgroups in our study revealed that older adults (over 60 years old) are most susceptible to the negative impacts of air pollution. Older women tolerate ozone air pollution less well than older men. As ozone concentrations increase above 100 μg/m^3^, the incidence of community-acquired pneumonia among women aged over 60 increases by an average of 22% compared to periods with low concentrations. For men in this age group, the same ozone concentrations increase the incidence of community-acquired pneumonia by an average of 14%. It is noteworthy that the safe level of ground-level ozone concentration for older adults is significantly lower than the WHO standard. These findings are consistent with the results of previous studies. Thus, the multicenter study conducted in four hospitals in four China regions [18] found a direct relationship between ground-level ozone and bacterial pneumonia morbidity rate, with a correlation coefficient of 0.41. Kwas et al. [34] studied the effect of outdoor air pollution on the severity and outcomes of CAP in the Gabes region, Tunisia, and showed significant relationships between air quality and weather conditions and various clinical scores for patients with pneumonia, though the authors failed to find a significant correlation between ozone and clinical scores for CAP. Wang et al. [35] assessed the short-term impact of air pollution on hospitalization of residents of Qingdao (China) with pneumonia and identified vulnerable groups. The results of their study showed that the events of short-term increase in ground-level ozone concentrations were associated with an increase in the cases of hospitalizations with pneumonia in the city residents as compared to the rural population.

Lu et al. [36] examined the short-term effects of ambient air pollution on the number of hospitalizations for pneumonia and found significant positive correlations between ozone and hospitalizations for pneumonia with a time lag of 4 days for COPD patients aged 60 years and over. The above observations show that some people are particularly susceptible to this oxidizing gas.

## 5. Conclusions

The results of our study indicate that the increased ozone concentration in the atmospheric surface layer in the summer period in Moscow leads to the increased number of cases of community-acquired pneumonia among Moscow residents. Older people (over 60 years old) are particularly vulnerable to the negative impact of atmospheric pollution. Older women are found to be more sensitive to ozone pollution than men of this age group. Furthermore, older women may experience adverse health effects at ozone concentrations lower than those recommended by the WHO. Young people are virtually immune to ozone concentrations within the permissible limits (up to 150 μg/m^3^). To sum up, we would like to note the measures that should be taken in cities at the state level in this regard, such as, for example:

1. Organizing a network for the permanent monitoring of near-surface ozone concentration in large cities;
2. Developing early warning systems for the population regarding increased ozone concentrations;
3. Conducting an information campaign on the risks associated with ozone pollution to raise the awareness of residents about the negative impact of this pollution on health and ways to reduce it.

## Figures and Tables

**Table 1:** Daily averages of summer average values of parameters.

Year	O_3__MDA8, µg/m^3^	t_max_, °C	CAP, Cases/Millions Persons
All	70.52	24.18	9.17
2006	57.45	22.9	8.28
2007	79.62	24.05	8.51
2008	65.65	22.26	9.41
2009	57.13	22.05	9.46
2011	92.75	25.94	10.21

**Table 2:** Time dependence of the cross-correlation coefficient between CAP and ground-level ozone concentration or maximum air temperature.

Lag, Day	r (CAP, O_3__MDA8)	r (CAP, t_max_)
0	0.47	0.38
1	0.51	0.42
2	0.51	0.41
3	0.46	0.33
4	0.37	0.24
5	0.29	0.15
6	0.23	0.08
7	0.18	0.03

## Data Availability

The datasets analyzed in the current study are available upon formal request from the following organizations: Mosecomonitoring https://mosecom.mos.ru/ (accessed on 14 January 2026); Russian Federal State Statistics Service https://www.fedstat.ru/indicator (accessed on 14 January 2026); Urgent Medical Care Station of the Moscow Department of Health https://mos03.ru/ (accessed on 14 January 2026).

## Abbreviations

The following abbreviations are used in this manuscript:

WHO	World Health Organization
AQG	Air Quality Guideline
COPD	Chronic obstructive pulmonary disease
CAP	Community-acquired pneumonia
MDA8	Daily maximum 8 h average
